# Indirect Estimation of 25-Hydroxyvitamin D Reference Intervals Using Data Mining

**DOI:** 10.3390/diagnostics16101436

**Published:** 2026-05-08

**Authors:** Esra Yılmaz, Hülya Kılıç

**Affiliations:** 1 Clinical Biochemistry Laboratory, Karabük Training and Research Hospital, Karabuk 78200, Türkiye; trn6325@gmail.com; 2 Department of Medical Biochemistry, Faculty of Medicine, Recep Tayyip Erdogan University, Rize 53100, Türkiye

**Keywords:** 25-hydroxyvitamin D, data mining, reference range, reference value

## Abstract

**Background:** Reference intervals for 25-hydroxyvitamin D are significantly influenced by seasonal variations and population-specific factors. This study aimed to establish local, population-specific Vitamin D reference intervals using the refineR algorithm and to compare these results with the conventional non-parametric method using a hospital database. **Methods:** A total of 127,220 laboratory results from 2022 to 2025 were retrospectively analyzed. Data were filtered based on adult age, morning fasting specimens, outpatient status, and normal parathyroid hormone levels (14–72 ng/L). Following filtration, Wilcoxon and Kruskal–Wallis tests were employed to evaluate differences between potential subgroups. Consequently, 24,036 eligible results were stratified by age (18–40, 41–65, and >65 years) and sex (female and male). Reference intervals were calculated using the refineR algorithm and the non-parametric percentile method. **Results:** The distribution of vitamin D levels was found to be right-skewed [Median: 12.92 ng/mL (4.21–149)]. The upper reference limits obtained via refineR were consistently lower than those derived from the non-parametric method. Both methods showed a linear increase in median values and upper reference limits with advancing age. Females in the 18–40 age group exhibited the lowest vitamin D profile. The summer concentrations were significantly higher than those of other seasons (*p* <0.05). **Conclusions:** The refineR algorithm managed outliers and pathological results more effectively than the non-parametric method. Our findings highlight the clinical necessity for age- and sex-specific subgroups, rather than relying on the manufacturer’s single reference range. Implementing these population-specific intervals can enhance diagnostic accuracy and prevent misclassification, facilitating earlier identification of vitamin D-related metabolic and skeletal disorders.

## 1. Introduction

Accurate laboratory result interpretation depends on appropriately defined reference intervals (RIs) [1]. While the Clinical and Laboratory Standards Institute (CLSI) recommends population-specific RIs [2], many laboratories utilize manufacturer-provided or transferred intervals due to the high cost and logistical challenges of direct determination. RIs can be established via direct methods (selecting healthy individuals) or indirect methods [statistically analyzing Laboratory Information System (LIS) datasets]. Indirect methods have gained prominence recently as cost-effective alternatives [3], with support from the International Federation of Clinical Chemistry and Laboratory Medicine Committee on Reference Intervals and Decision Limits (IFCC C-RIDL) [1].

Vitamin D is a critical parameter in mineral metabolism and systemic health [4]. Status is typically assessed via 25-hydroxyvitamin D (Vitamin D) or 1,25-dihydroxyvitamin D. Although 1,25-dihydroxyvitamin D is the active hormone, its short half-life, tight regulation, and analytical challenges (e.g., picomolar concentrations and method variability) limit its routine use to specific conditions like chronic kidney disease [5,6,7]. Conversely, Vitamin D is analytically robust, with higher concentrations and longer half-life, making it the preferred analyte for RI studies using modern immunoassays or liquid chromatography–tandem mass spectrometry [8,9]. Although inter-method variability between immunoassays and LC-MS/MS remains a technical challenge for the universal application of standardized RIs, international efforts such as the Vitamin D Standardization Program have significantly improved the traceability and comparability of results across different analytical platforms. Serum Vitamin D is a key indicator of bone metabolism. Deficiency is linked to skeletal, oncological, cardiovascular, and autoimmune disorders [8,10,11,12,13], though supplementation benefits remain debated. Despite its global deficiency prevalence, universal Vitamin D RIs are lacking [14]. Transferred RIs may lead to misinterpretation, especially for parameters influenced by seasonal sunlight exposure, necessitating geographically specific intervals. Furthermore, the 2024 Endocrine Society guideline underscored the distinction between RIs and clinical decision limits, refraining from recommending uniform thresholds for the general population [15].

Laboratory information system datasets now facilitate indirect RI estimation using predefined criteria. Among available tools, the refineR algorithm offers superior reproducibility and user-independence compared to traditional Hoffman or Bhattacharya methods. Notably, refineR maintains high accuracy even when pathological results comprise up to 30% of the dataset [16].

In this study, we established population-specific Vitamin D RIs using the refineR algorithm. Through rigorous data preprocessing and statistical modeling, we provide a robust comparison with conventional non-parametric methods. These intervals offer a practical framework for improving the clinical interpretation of laboratory reports in our region.

## 2. Materials and Methods

In this study, 127,220 serum Vitamin D results conducted in our laboratory between 1 January 2022 and 1 January 2025 were analyzed. Following data filtering which included the exclusion of results from inpatient and emergency departments, non-fasting samples, repeat measurements and individuals with abnormal PTH levels reference intervals were calculated using the nonparametric method and the refineR algorithm (version 1.6.1). This retrospective study was approved by the local ethics committee (Decision No: 2025/427, Date: 9 October 2025). Due to the retrospective and de-identified nature of the data, the requirement for informed consent was waived by the committee.

Blood samples were collected into serum separator tubes (Vacusera, Disera Medical Supplies, İzmir, Türkiye), allowed to clot for 20 min, and centrifuged at 3000× *g* for 10 min. Samples were analyzed within 3 h of collection. The Vitamin D analysis was performed using a chemiluminescent immunoassays reagent on the Siemens Advia Centaur XPT platform (Siemens Healthineers, Erlangen, Germany).

Initially, 127,220 results from 27,976 unique individuals were identified. Inclusion was restricted to individuals aged ≥18 years. To ensure a representative healthy subpopulation, samples from emergency departments, inpatient wards, and subspecialty clinics were excluded. Furthermore, only morning fasting specimens (collected between 08:00 and 12:00, *n* = 25,054) and the earliest available result for each individual were included to minimize the confounding effects of vitamin D supplementation leaving 24,856 participants. Additionally, patients with PTH levels outside the reference interval (14–72 ng/L) were excluded (*n* = 820). After filtering, 24,036 eligible results were categorized by sex, age, season, and clinical unit.

Internal quality control (IQC) and external quality control (EQC) performances were evaluated for 25 hydroxyvitamin D measurements. Internal quality control data were obtained using Randox control materials (Randox Laboratories, Crumlin, UK), and the combined coefficient of variation (%CV) was calculated [17]. External quality control performance was monitored through the Randox International Quality Assessment Scheme (RIQAS) immunoassay program, and bias values were derived from monthly reports. In our laboratory, a total of 665 level 1 and 794 level 2 IQC results obtained from two different analyzers used for 25 hydroxyvitamin D analysis were evaluated, and the overall %CV was calculated based on these data. In addition, bias (%) values were derived from external quality control results collected over a two-year (24 months) period. Total analytical error (TAE) was calculated using the formula: %TAE = %Bias + 1.65 × %CV. In this calculation, 1.65 represents the one-sided z-score corresponding to a 95% confidence level. The analytical performance indicators determined for Vitamin D measurements in our laboratory were a CV of 7.8%, a bias of 11.5%, and a TAE of 24%.

The dataset was categorized by age (18–40, 41–65, and >65 years), sex (female and male), season [Autumn (September–October–November), Winter (December–January–February), Spring (March–April–May), and Summer (June–July–August)] and requesting clinical unit (Family Medicine, Physical Medicine and Rehabilitation, Internal Medicine, Orthopedics and Other). Wilcoxon and Kruskal–Wallis tests were employed to evaluate differences in median Vitamin D concentrations between potential subgroups. The dataset was stratified by age and sex. Reference intervals (RIs) were established using the refineR algorithm and the nonparametric percentile method. Prior to the nonparametric analysis, outliers were identified and excluded using Tukey’s interquartile range (IQR) method to prevent distortion. Each subgroup met the minimum sample size requirement *n* ≥ 120. Finally, 95% confidence intervals for the estimated reference limits were calculated using the bootstrap method. For the refineR analysis, the findRI function was applied directly to the filtered dataset without any prior manual outlier exclusion or data transformation, as the algorithm is designed to handle these processes automatically. To account for the highly skewed nature of Vitamin D distribution, the internal mod Box–Cox transformation was utilized to model a latent Gaussian distribution. The RIs and their 95% confidence intervals were estimated using 200 bootstrap iterations within the refineR framework.

Data storage and initial preprocessing were managed using Microsoft Excel (Microsoft 365, Microsoft Corp., Redmond, WA, USA). Detailed data manipulation, statistical modeling and indirect reference interval estimations were performed using the refineR algorithm within the R software (version 4.4.2, R Foundation for Statistical Computing, Vienna, Austria) and RStudio (version Cranberry Hibiscus, Posit Software, PBC, Boston, MA, USA). A *p*-value less than 0.05 was considered statistically significant. All high-resolution visualizations were generated using the R statistical environment.

## 3. Results

### 3.1. Data Summary

The reference sample comprised 6056 men and 17,980 women. The ages of the participants ranged from 18 to 99 years, with a median age of 50 years.

**Figure 1:**
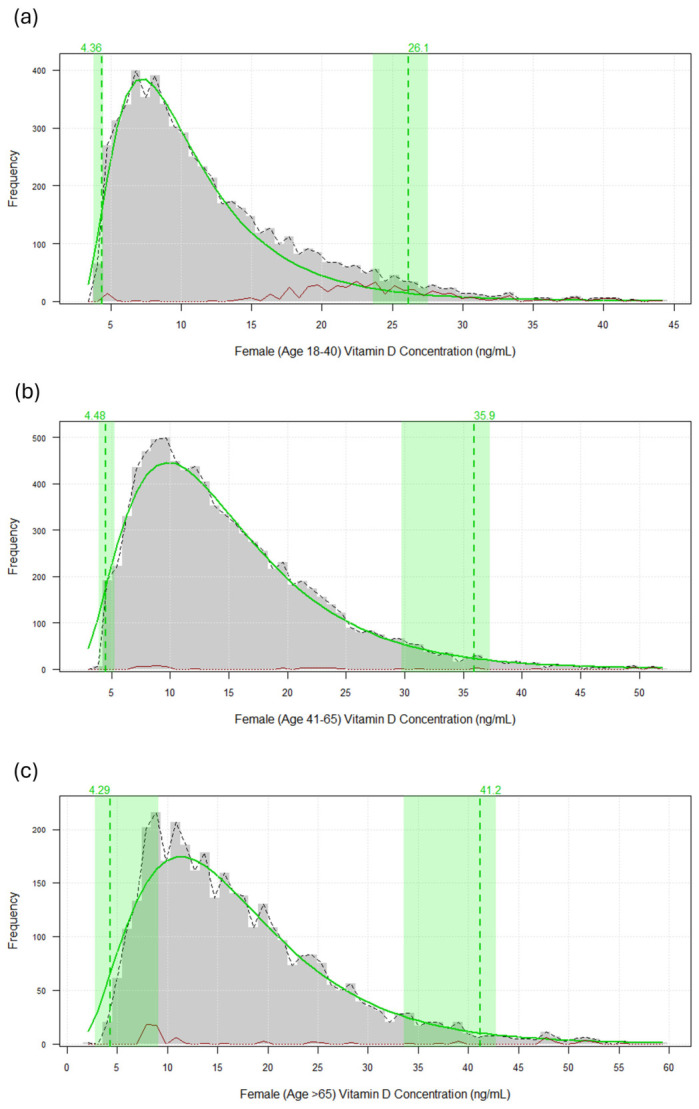
Estimated reference intervals for female vitamin D concentrations across different age groups using the refineR algorithm. Description of the estimated underlying distribution and 95% reference interval for females aged 18–40 years (**a**); for females aged 41–65 years (**b**); for females aged >65 years (**c**). The grey histograms represent the observed frequency of 25-hydroxyvitamin D concentrations (ng/mL). The solid green line indicates the modeled healthy distribution, while the dashed green vertical lines and shaded areas denote the calculated lower and upper reference limits. The dashed black line represents the observed distribution of the raw data. The red line at the baseline illustrates the identified pathological or non-representative distribution components partitioned by the algorithm.

Following the application of inclusion and exclusion criteria, the original Vitamin D concentrations exhibited a significantly right-skewed distribution (Figure 1), with a median level of 12.92 ng/mL (4.21–149.04 ng/mL).

The median and IQR of Vitamin D levels for each subgroup are detailed in Table 1.

The total analytical error for Vitamin D analysis was calculated as 24%, which is within the acceptable performance limits (±25%) established by the 2024 CLIA proficiency testing criteria. This indicates that the analytical performance of the Siemens Advia Centaur XPT system meets current international regulatory standards for clinical reliability.

### 3.2. Subgroup Analysis and Calculation of Reference Intervals

Vitamin D levels varied significantly across sexes (Wilcoxon, *p* < 0.001) and were further influenced by age, season, and clinical unit (Kruskal–Wallis, all *p* < 0.001). Bonferroni-corrected pairwise comparisons identified significant differences between most age groups (18–40, 41–65, and 66–80; *p* < 0.001), except between the 66–80 and >80 cohorts (*p* = 1.00). Seasonally, summer concentrations were highest (*p* < 0.001), while autumn represented an intermediate phase. No significant difference was observed between winter and spring (*p* = 1.00). Physical Medicine and Rehabilitation results differed significantly from others (*p* < 0.001). Although these variables warrant consideration for local reference intervals, this study focused on age and sex stratification to maintain the minimum sample size (*n* > 400) required for the refineR algorithm to provide reliable estimates. The reference intervals and their 95% confidence intervals (CIs) calculated via the refineR algorithm are presented in Table 2.

The results from the refineR algorithm and the non-parametric method were very close to each other, although their exact limits differed slightly. While both methods captured a clear age-related upward trend in Vitamin D levels, the refineR-derived RIs provided slightly more conservative upper limits in specific cohorts, likely due to its ability to minimize the influence of outlier distributions inherent in hospital-based data. Despite these minor algorithmic variations, the lower limits remained consistently low across both methods (range: 4.3–5.8 ng/mL in Table 2 vs. 4.6–6.5 ng/mL in Table 3), confirming that the choice of statistical approach does not alter the fundamental finding of profound Vitamin D deficiency in the region.

**Figure 2:**
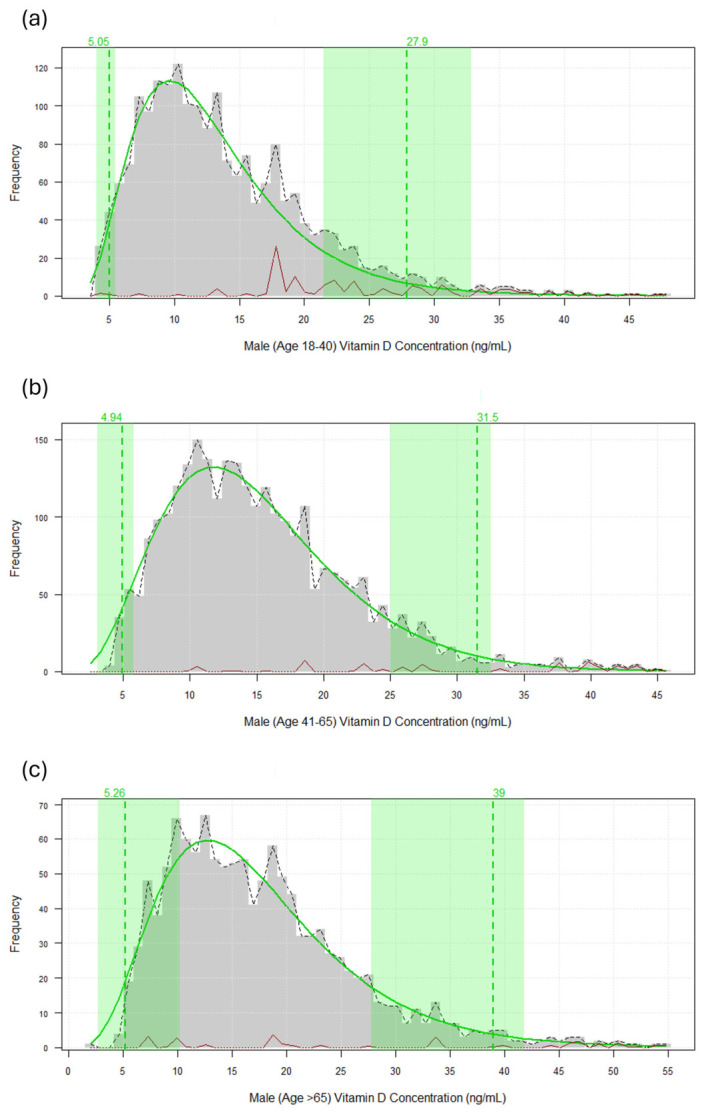
Estimated reference intervals for male vitamin D concentrations across different age groups using the refineR algorithm. Description of the estimated underlying distribution and 95% reference interval for males aged 18–40 years (**a**); for males aged 41–65 years (**b**); for males aged >65 years (**c**). The grey histograms represent the observed frequency of 25-hydroxyvitamin D concentrations (ng/mL). The solid green line indicates the modeled healthy distribution, while the dashed green vertical lines and shaded areas denote the calculated lower and upper reference limits. The dashed black line represents the observed distribution of the raw data.The red line at the baseline illustrates the identified pathological or non-representative distribution components partitioned by the algorithm.

The reference intervals derived from the refineR algorithm, including the visual representation of the estimated underlying distributions, are illustrated in Figure 1 for female-age-specific results and Figure 2 for male-age-specific results.

## 4. Discussion

In this study, a remarkable alignment and key methodological differences were identified between the traditional Tukey-based non-parametric method and the modern indirect modeling approach, the refineR algorithm. The finding that lower reference limits (LL) remained consistently between 4.3 and 6.5 ng/mL across both models confirms that severe vitamin D deficiency in this population is a stable biological finding, independent of the statistical method used.

In contrast, the refineR model produced more conservative and lower values for the upper reference limits (UL) compared to the non-parametric method. For instance, in the male group aged 18–40, the UL of 32.01 ng/mL determined by the non-parametric method decreased to 27.90 ng/mL with the refineR model. This suggests that the refineR algorithm more effectively partitions high values associated with exogenous vitamin supplementation or pathological processes, thereby providing a more biologically representative reference interval.

Analysis of subgroup trends revealed a significant linear increase in both median values and upper limits with advancing age. Specifically, the rise in the upper limit from 26 ng/mL in younger groups to the 41 ng/mL range in individuals aged 65 and over may reflect differences in bone metabolism or more prevalent supplement use in this demographic. Gender-based analysis showed that males generally maintain a higher vitamin D profile than females, with the 18–40 age group of females emerging as the subset with the lowest values in the entire population across both methods. Furthermore, the width of the 95% Confidence Intervals (95% CI) provided critical insights into data precision; the narrow CIs in the 18–40 female group confirm the statistical certainty of the deficiency in this cohort, while the relatively wider intervals in males over 65 symbolize higher heterogeneity and individual variation. Ultimately, while the Tukey method provides a statistical reflection of the current hospital population, the refineR method offers a more reliable estimation closer to the true biological reference range by minimizing latent pathological distributions.

While indirect methods offer advantages over the practical difficulties of direct methods, poor management of raw data can lead to erroneous estimates. Literature indicates that direct and indirect methods may yield disparate results. For example, a study conducted in Spain reported an indirect RI of 5.57–57.05 ng/mL, whereas the direct RI obtained from healthy volunteers was 8.98–41.27 ng/mL. This discrepancy arises because some indirect methods may incorporate widespread population deficiency into the model as part of the normal distribution. Although refineR serves as a sophisticated and robust alternative, its accuracy remains dependent on the quality and size of the dataset.

### 4.1. Effects of Sex, Age, and Season

Subgroup analyses demonstrated that vitamin D levels are significantly influenced by sex, age, and season. Consistent with the high prevalence of deficiency reported in the current literature, the refineR algorithm defined lower upper limits for females and outperformed the non-parametric approach in capturing the biochemical characteristics of the local population. Additionally, the lower upper limits observed in females compared to males suggest either more widespread supplement use among males or, conversely, lower sunlight exposure among females. These findings align with existing literature; for example, Oğuzman and Doğan reported vitamin D deficiency is 67.5% of females and 54.9% of males [18]. If a fixed clinical threshold, such as 20 ng/mL, had been applied in our study, the female population would have exhibited a markedly higher rate of deficiency. These findings underscore that geographic and population-specific factors—such as dietary habits, clothing styles, and sunlight exposure—limit the universal applicability of manufacturer-provided reference intervals, highlighting the necessity of establishing laboratory-specific local ranges.

Factors such as limited sunlight exposure due to traditional clothing styles and differences in dietary habits likely contribute to these lower levels in women [19]. To minimize the potential confounding effect of vitamin D supplementation, only the results from the initial visit were included in the analysis. Our study revealed that the reference intervals obtained via the refineR algorithm were significantly higher in the elderly age group compared to the younger cohorts; this trend remained consistent across both calculation methods.

The elevated Upper Reference Limit (URL) observed in the elderly population may reflect a high vitamin D clustering effect. This phenomenon is likely attributable to more intensive vitamin D supplementation in older individuals or closer clinical monitoring and management within the scope of chronic disease follow-up [20]. On the other hand, despite the elevated trend in the upper limits, it is noteworthy that the lower reference limits (LRL) obtained via refineR exhibited wider confidence intervals and lower absolute values. These lower LRLs observed in the refineR method appear to be more sensitive than the non-parametric approach in reflecting physiological synthesis deficiencies and reduced sunlight exposure prevalent in the elderly population. This suggests that the refineR algorithm is more successful in modeling the heterogeneous structure and subgroup-specific biochemical deficiencies inherent in the advanced age population. However, the effect of age can vary by region; for instance, some studies in Türkiye have reported no significant age-related differences in vitamin D concentrations [21,22,23].

As expected, seasonal variations were clearly reflected in the study results; vitamin D levels detected during the summer months were significantly higher than those in other seasons. Post hoc analyses revealed no statistically significant difference between the winter and spring periods, whereas autumn results differed significantly from all other seasons.

However, it was determined that including seasonal variables in the subgroup analyses would reduce the sample size below the minimum requirement (N) for the reference interval calculation methods. Therefore, to maintain the representativeness of the subgroups and ensure statistical reliability, a season-based stratification was not pursued for the final reference interval estimations. Consequently, it is observed that seasonal factors, alongside sex and age variables, significantly influence 25 hydroxyvitamin D levels; thus, these demographic variables must be integrated into the clinical interpretation process of laboratory results [14].

In addition to age, sex, and seasonal variations, we observed that vitamin D levels in the PMR department were significantly higher than in other major units. This finding likely reflects the high clinical awareness within this specialty regarding bone health and osteoporosis management. Patients followed in PMR clinics are more frequently screened for deficiency and are often under active supplementation or regular monitoring, which shifts the departmental distribution toward higher values. This observation underscores the importance of considering clinical context when interpreting laboratory data.

### 4.2. Differences from Manufacturer Reference İntervals and Clinical İmplications

The laboratory-specific 25 hydroxyvitamin D reference intervals established in this study differed markedly from those provided by the manufacturer. The manufacturer recommends a general reference interval of 7.4–44 ng/mL. In contrast, most of the reference intervals calculated using refineR in our subgroups were lower than the manufacturer’s range. Similarly, M. Alpdemir and MF. Alpdemir reported a local reference interval of 6.45–30 ng/mL (2.5th–97.5th percentiles) in a study conducted in the Balıkesir region using data from 2010 to 2014, which was significantly lower than the manufacturer-provided interval [24]. Likewise, Oğuzman and Doğan demonstrated a lack of concordance between manufacturer reference intervals and local population data in a study based on data from the Hatay region collected between 2018 and 2019 [18]. These findings indicate that geographic and population-specific differences limit the universal applicability of manufacturer-provided reference intervals, highlighting the importance of establishing local reference ranges.

Manufacturers typically derive reference intervals from a limited number of healthy volunteers or from generalized literature data. However, such intervals may not be appropriate for local populations owing to differences in sunlight exposure, dietary habits, and genetic background across geographic regions. Therefore, laboratories should validate manufacturer reference intervals in their own patient populations or redefine them when necessary. Both the IFCC and CLSI recommend that laboratories establish reference intervals specific to their analytical methods and populations when suitable population-based references are unavailable [14,25,26]. Our findings further support the need to reassess manufacturer-provided 25–hydroxyvitamin D reference intervals in Türkiye. Potential reasons for these discrepancies including methodological bias and widespread vitamin D deficiency should be clearly communicated to clinicians. Applying manufacturer-derived intervals to populations may lead to misclassification and delayed intervention. Our findings suggest that population-specific reference intervals improve diagnostic sensitivity by accounting for local status. This is particularly relevant for high-risk groups, such as older adults, where accurate Vitamin D assessment is critical for managing musculoskeletal and cardiovascular outcomes. Localized intervals thus offer a more tailored approach to clinical risk stratification.

### 4.3. Distinction Between Reference İntervals and Clinical Decision Limits

In its 2018 position statement, the IFCC C-RIDL emphasized the importance of clearly distinguishing reference intervals from clinical decision limits and highlighted the active role of laboratory specialists in the appropriate application of both concepts [27]. Significant changes have occurred in the clinical interpretation of vitamin D concentrations in recent years. Unlike the 2011 version, the 2024 Endocrine Society guideline refrains from defining a fixed “normal” serum 25-hydroxyvitamin D concentration for the healthy population, recommends limiting routine vitamin D screening, and suggests that moderate-dose supplementation is generally sufficient [15]. The updated guideline moves away from categorical classifications such as deficiency (<20 ng/mL) and insufficiency (20–30 ng/mL) and instead focuses on recommended dietary allowance-based intake in at-risk groups, including older adults, pregnant women, individuals with prediabetes, and children. It also emphasizes evidence showing no clear benefit of unnecessary testing or high-dose supplementation in healthy adults [15]. This evolving approach illustrates that clinical decision limits may change over time in response to emerging scientific evidence. Accordingly, laboratory physicians should consider current clinical decision thresholds alongside reference intervals when reporting results.

### 4.4. Limitations

This study has several limitations, primarily the lack of information on key confounders such as sunlight exposure, BMI, and vitamin D supplementation, as the latter is not systematically recorded in the LIS. While reliability was supported by excluding individuals with abnormal PTH levels, the relatively small subset of patients with available PTH data (*n* = 820) may limit the generalizability of this specific filtration. Consequently, these unmeasured variables could influence the upper boundaries of the reference intervals, highlighting the need for future prospective studies to incorporate broader biochemical markers for a more precise estimation of the healthy population.

## 5. Conclusions

The refineR algorithm provided a robust and sensitive approach to managing extreme values in laboratory data, outperforming traditional non-parametric methods in this context. Our findings indicate that sex, age, and seasonality significantly influence 25-hydroxyvitamin D concentrations, suggesting that universal reference ranges provided by manufacturers may not fully reflect the biochemical characteristics of local populations. Therefore, adopting population-specific reference intervals has the potential to minimize the misclassification of Vitamin D status and support the early detection of deficiency-related metabolic risks.

## Figures and Tables

**Table 1:** Distribution of 25–hydroxyvitamin D concentrations according to demographic and clinical subgroups.

Subgroup (n)	Median (Q1–Q3), ng/mL
Age 18–40 (7904)	10.8 (7.7–16.1)
Age 41–65 (11,361)	13.5 (9.5–19.2)
Age 66–80 (3939)	15.4 (10.5–21.7)
Age 80+ (832)	15.4 (9.7–22.7)
Female (17,980)	12.5 (8.6–18.6)
Male (6056)	13.9 (10.0–19.3)
Autumn (6364)	14.6 (10.3–20.4)
Winter (6431)	11.4 (8.0–17.3)
Spring (5511)	11.3 (8.0–17.1)
Summer (5730)	14.2 (9.9–19.8)
PMR * (6810)	14.9 (10.3–20.9)
IM ** (16,377)	12.1 (8.4–17.8)
Orthopedics (267)	14.1 (9.1–20.0)
FM ^#^ (59)	12.1 (8.9–14.7)

Q1: First quartile, Q3: Third quartile, * Physical Medicine and Rehabilitation, ** Internal Medicine, ^#^ Family Medicine.

**Table 2:** The reference intervals and their 95% confidence intervals (CI) calculated via the refineR algorithm.

Sex (Age)	N	Median (Min–Max)	Lower Limit (95% CI)	Upper Limit (95% CI)
Male (18–40)	1993	12.6 (2.1–64.6)	5.1 (4.0–5.5)	27.9 (21.5–32.8)
Male (41–65)	2813	14.2 (4.2–68.7)	4.9 (3.1–5.8)	31.5 (25.0–32.5)
Male (65+)	1250	16.1 (2.1–82)	5.3 (2.8–10.2)	39.0 (27.8–41.8)
Female (18–40)	5911	10.2 (2.1–149.0)	4.4 (3.8–4.5)	26.1 (23.6–27.6)
Female (41–65)	8548	13.2 (2.1–106.0)	4.5 (3.9–5.3)	35.9 (29.7–37.3)
Female (65+)	3521	15.1 (2.1–108.0)	4.3 (2.8–9.1)	41.2 (33.5–42.8)

The non-parametric percentile method results and their 95% confidence intervals (CIs) are shown in Table 3.

**Table 3:** The reference intervals and their 95% confidence intervals (CI) calculated via nonparametric percentile method.

Sex (Age)	N	Median (Min–Max)	Lower Limit (95% CI)	Upper Limit (95% CI)
Male (18–40)	1992	12.6 (4.2–56.5)	5.1 (4.9–5.3)	32.0 (30.3–34.4)
Male (41–65)	2803	14.2 (4.5–54.2)	5.6 (5.4–6.0)	34.4 (32.4–36.9)
Male (65+)	1245	16.1 (4.6–67.7)	6.5 (6.1–6.8)	40.1 (37.7–44.7)
Female (18–40)	5900	10.2 (4.2–55.2)	4.6 (4.6–4.7)	30.2 (29.1–31.3)
Female (41–65)	8539	13.2 (4.2–66.1)	5.1 (5.0–5.3)	36.1 (35.1–36.9)
Female (65+)	3518	15.0 (4.2–82.0)	5.6 (5.4–5.8)	42.6 (40.0–45.2)

## Data Availability

The datasets generated and analyzed during the current study are not publicly available due to institutional regulations and patient privacy restrictions. However, the data are available from the corresponding author on reasonable request and with the permission of the relevant institutional ethics committee.
